# Letter from the Editor in Chief

**DOI:** 10.19102/icrm.2020.110906

**Published:** 2020-09-15

**Authors:** Moussa Mansour


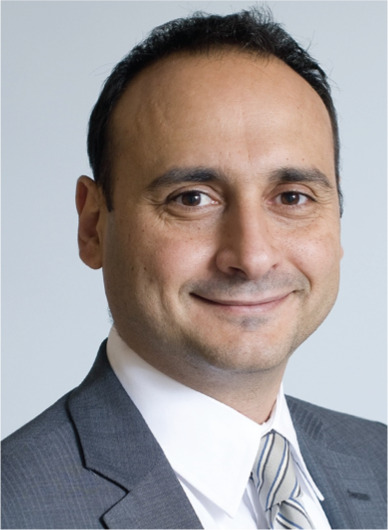


Dear Reader,

At the time of writing this, the 2020 scientific meeting of the European Society of Cardiology (ESC) is currently underway in a virtual format. To date, the meeting has been rich with studies related to atrial fibrillation (AF), including, in particular, two major randomized multicenter studies: Early Rhythm-control Therapy in Patients with AF (EAS-AFNET 4) and Cryoballoon Catheter Ablation in Antiarrhythmic Drug-naive Paroxysmal AF (STOP AF First).^1^

In EAST-AFNET 4, a landmark study presented as a late-breaking clinical trial with simultaneous publication in *The New England Journal of Medicine,*^2^ 2,789 patients with early AF and other cardiovascular conditions were randomized to either early rhythm control or rate control. Two primary endpoints were considered: (1) a composite of death from cardiovascular causes, stroke, or hospitalization with worsening of heart failure or acute coronary syndrome and (2) the number of nights spent in the hospital. The primary safety outcome was a composite of death, stroke, or other treatment-related serious adverse events. During follow-up, the early rhythm-control strategy proved superior regarding both primary endpoints; interestingly, in the first two years, just 19% of patients in the rhythm-control group underwent ablation, while antiarrhythmic medications were given to the remaining majority. There was no significant difference in the rate of adverse events between the two groups.

Separately, STOP AF First compared cryoballoon ablation and antiarrhythmic drug therapy as first-line treatments in 225 patients with paroxysmal AF followed for two years. Freedom from AF was achieved in 75% of patients undergoing ablation but in 45% in the medication group. Most importantly, ablation was associated with a very low rate of complications (1.9%).

The results of these studies come one year after the ESC’s presentation of the Atrial Fibrillation Progression Trial (ATTEST),^3^ where patients treated with catheter ablation were almost 10 times less likely to develop persistent AF than those on antiarrhythmic drugs. These three studies are complementary and, when paired with novel advanced ablation technologies, likely to drive a shift toward performing ablation earlier on in the course of AF.

Sincerely,


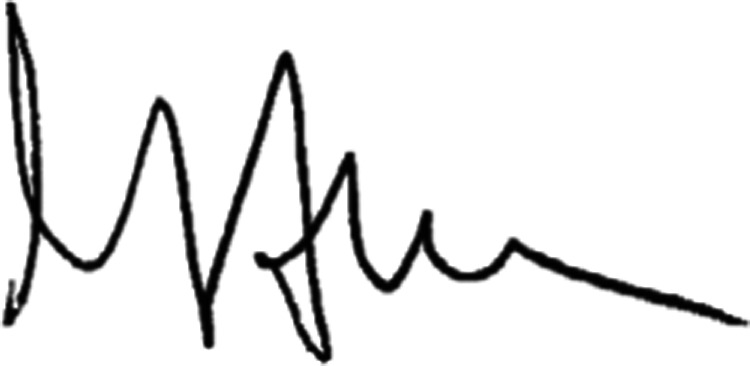


Moussa Mansour, MD, FHRS, FACC

Editor in Chief

The Journal of Innovations in Cardiac Rhythm Management

MMansour@InnovationsInCRM.com

Director, Atrial Fibrillation Program

Jeremy Ruskin and Dan Starks Endowed Chair in Cardiology

Massachusetts General Hospital

Boston, MA 02114
